# Supplementary figures and images for: Land reclamation and its consequences: A 40-year analysis of water residence time in Doha Bay, Qatar
Source: PLoS One. 2024 Jan 31;19(1):e0296715. doi: 10.1371/journal.pone.0296715 (PMC10829981; doi:10.1371/journal.pone.0296715)

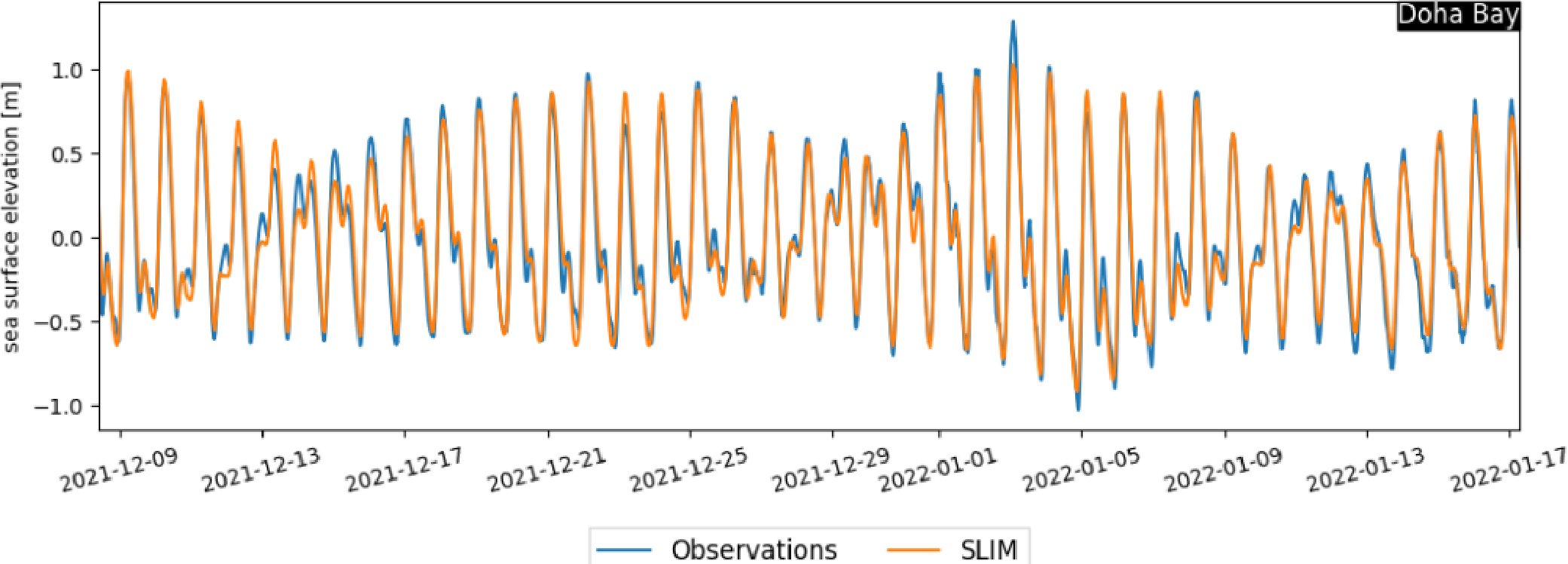

Supplement: S1 Fig — Although the model we employ has previously been validated for sea surface elevation and current velocity within Doha Bay and northeast of Qatar, we had the opportunity to access additional sea surface elevation measurements from the center of the Bay (25°19’48.0“N, 51°33’36.0“E) taken between December 8, 2021, and January 17, 2022. This period aligns with the winter season simulated in our model. While the model’s generated sea surface elevation exhibits a slightly smaller magnitude than the observed data, the simulation closely mirrors the actual observations. The root mean square error (RMSE) between the simulated and observed elevations amounts to 9.6 cm. This result also indicates that the tidal amplitude within the Bay exceeds one meter. Given the Bay’s limited water depth, this suggests that the flow within the Bay is predominantly influenced by the tides. (TIF) [file pone.0296715.s001.tif]
