# Supplementary material for: Land reclamation and its consequences: A 40-year analysis of water residence time in Doha Bay, Qatar
Source: PLoS One. 2024 Jan 31;19(1):e0296715. doi: 10.1371/journal.pone.0296715 (PMC10829981; doi:10.1371/journal.pone.0296715)
Supplement: Attachment — Submitted filename: response2reviewer.pdf [file pone.0296715.s002.pdf]

Response to the reviewers' comments on the paper "Land reclamation and its consequences: A 40-year analysis of water residence time in Doha Bay, Qatar" submitted to the *PLOS ONE*

Emmanuel Hanert, on behalf of all coauthors

November 30, 2023

Dear Dr Tariq Umar,

We would like to thank you and both reviewers for dedicating time to meticulously review our paper. We greatly value the reviewers' feedback and found their comments instrumental in improving our manuscript. We have tried to implement all the suggestions provided in their thorough reviews as shown herein in the point-by-point response. We also respond to the different journal requirements that were included in your email.
In what follows, the reviewers' comments are presented in *italic* type, and our response in roman type. Changes to the manuscript are highlighted in *green* in this document and in track-change in the revised manuscript. Note that the line numbers indicated in our response refer to the track-change version of the revised manuscript.

Reviewer #1

(G) *The research is related to "Land reclamation in Doha Bay, Qatar and its consequences by conducting water residence analysis," a unique and possesses the potential to add a valuable understanding of the problem specific to Doha Bay, Qatar. However, here are some comments for the authors. They should consider these suggestions to enhance the quality and impact of their research.*

Response: Thank you for your positive appreciation of our work! We are grateful for your constructive comments and suggestions. We have carefully considered each of your recommendations and have made concerted efforts to incorporate them into our revised manuscript.

(1) *Introduction: The section is generally well-written and provides a clear introduction to coastal alterations in the Gulf due to rapid development. Figure1: The review suggests adding numbers or indications to the locations mentioned in Figure 1, which would help readers identify them easily. There's a recommendation to replace the word "red" with "pink" in the description of the wastewater outfalls to match the figure's color.*

Response: Thank you for your suggestions regarding Figure 1. We now refer in the text to the location numbers mentioned in the figure (see lines 21, 24, 26, 81, 82 and 84). We have also changed the color name to magenta in the figure caption.

(2) *Adding references: The review suggests incorporating in-text references to support the claims made in the introduction. For example, adding a reference to "Water residence time is a simple diagnostic to assess water quality in a particular coastal area such as a bay, a lagoon or an estuary".*

Response: Thank you for your suggestion to strengthen the introduction with additional in-text references. We have carefully reviewed the section and have now incorporated new references to support our claims, particularly regarding the use of water residence time as a diagnostic tool for assessing water quality in coastal areas like bays, lagoons, or estuaries. New references to Fischer (1979), Takeoka (1984) and Monsen et al. (2002) can be found at lines 45-48 in the revised manuscript.

(3) *Clarify how the study will transition between the different periods (1980, 2000, and 2020) and the significance of these specific years in coastal development.*

Response: In our study, we have selected the years 1980, 2000, and 2020 as pivotal points to delineate the developmental phases of Doha Bay. As it is now better outlined in the introduction (see lines 60-68), the bay was relatively undeveloped prior to 1980, characterized primarily by its crescent-shaped coastline and the original port. The period from 1980 to 2000 marked a significant developmental shift, focusing primarily on the southern part of the Bay, highlighted by the expansion of the port and the construction of the new airport. Subsequently, from 2000 to 2020, development efforts were concentrated in the northern part of the Bay, evidenced by the emergence of The Pearl and Lusail City. Our study's approach is to analyze these three distinct years as representative milestones of Doha Bay's developmental trajectory: the initial phase of limited reclamation, followed by extensive reclamation in the southern part, and then a similar expansion in the northern part. This framework allows us to provide a focused yet comprehensive overview of the Bay's evolution, which would be challenging to achieve through a continuous chronological analysis. Our findings particularly highlight that the most recent phase of development, concentrated in the northern part of the Bay, has had the most pronounced impact on the water residence time.

The following text has been included: *We select these three distinct years as representative milestones of Doha Bay's developmental trajectory: the initial situation with limited reclamation, followed by extensive reclamation in the southern part of the Bay, and then a similar expansion in the northern part.*.

(4) *Results - Ocean circulation patterns: The authors should consider how this section connects with other parts of the paper, particularly the sections discussing water residence time and environmental impact. Ensure a smooth transition and integration of key findings and implications. If specific studies or literature influenced the analysis of circulation patterns, integrate citations in the text to support the findings.*

Response: In response to your feedback, we have revised and expanded the introductory paragraph of the Results section (see lines 185-195). This addition aims to more clearly articulate the interconnections between alterations in ocean circulation patterns, variations in water residence time, and their environmental impacts. Regarding the influence of prior studies on our analysis of circulation patterns, it is important to note that our research represents a pioneering effort in this specific area, as such investigations have not been previously conducted in Doha Bay. Consequently, direct citations pertaining to similar studies in the Results section are absent. Nevertheless, we added references to the relevant literature in the subsequent discussion section (see lines 319-323) and also in the introduction section (see lines 40-43).

The first paragraph of the Results section now reads: *We assessed the changes in water residence time between 1980, 2000 and 2020 by simulating the ocean circulation in the coastal area surrounding Doha Bay for the topographies of those three years. The ocean circulation in this area is predominantly tidally-driven, yet it is also influenced by wind patterns. These wind patterns exhibit seasonal variations, largely governed by the Shamal winds, which intensify during winter and subside in summer (Rao et al., 2003). We hence simulated ocean currents under both winter (01 December 2021 - 28 February 2022) and summer (01 June - 31 August 2022) wind conditions. These forcings were then maintained across all three topographies to isolate the influence of topographical changes on the ocean circulation and the subsequent impact on the residence time distribution.*

(5) *Results - Residence time patterns: Consider providing more detail on the reasons for the seasonal differences in residence time. Elaborating on these variations and offering potential explanations would strengthen the analysis. Mention if there are other studies or locations with similar findings to provide context and support the significance of the results. Are other regions facing similar challenges with artificial structures and their impact on residence time? Conclude the section by summarizing the key findings and their implications. How do these residence time patterns relate to the overall research question about the impact of land reclamation in Doha Bay?*

Response: In response to your comment, we now provide additional details on the reasons behind the observed seasonal variations. These variations are predominantly influenced by the shifts in the Shamal wind regime across the Gulf. We have elaborated on these changes in the revised introductory paragraph of the Results section (see lines 185-195 and transcript of the introductory paragraph of the Results section in our response to the previous comment). Please also note that we were already providing details on the wind patterns at the beginning of the Study Area section (see lines 91-96). It is further important to note that our study's primary aim is not to quantify the seasonal variations in ocean circulation offshore of Qatar in detail, as this aspect has already been covered in a previous study by Hanert et al. (2023). Instead, our focus is on showing that the changes induced by topographical modifications due to land reclamation overshadow the natural seasonal variability. Regarding the comparison with other studies or locations, we have reserved a comprehensive discussion of our results in the context of existing literature for the Discussion section. In this section, now enhanced, we delve deeper into how our findings reflect broader issues associated with extensive land reclamation projects, particularly those undertaken with limited environmental impact assessments (see lines 319-339 and our response to the next comment). This discussion aims to underscore the relevance of our study in understanding the implications of such developmental activities, thereby directly addressing the overarching research question concerning

the impact of land reclamation in Doha Bay.

(6) *Discussion and conclusions: The paper is encouraged to discuss potential mitigation measures. Highlighting recommendations for mitigating the environmental impact of development projects can enhance the paper's practical utility. Consider exploring potential solutions and strategies to address the issues raised in the paper. Offering guidance on balancing development with environmental preservation would be valuable. Suggest specific policy and regulation recommendations based on the research findings. How can environmental impact assessments be improved, or what regulations could mitigate the negative effects of land reclamation? Comparing the findings with similar studies in other regions, especially those with similar development projects, can provide context and make the results more generalizable. The authors must provide near-future recommendations given their results.*

Response: We really appreciate your suggestion to enhance the practical utility of our paper by discussing potential mitigation measures. In response, we have incorporated two additional paragraphs in the Discussion section, specifically dedicated to exploring viable strategies for mitigating the environmental impacts of artificial structures like those observed in our study. In this new paragraph, we not only discuss environmental impact assessment but also delve into recommendations for designing those structures. As requested by the second reviewer, we have also included a new paragraph that outlines the limitations of our model. This addition not only provides transparency regarding our study's scope but also offers insights into areas for future research.

The additional paragraphs read: *To mitigate the environmental impact of land reclamation projects in Gulf countries, a multifaceted approach that balances development with environmental preservation is essential. Enhancing the effectiveness of Environmental Impact Assessment (EIA) is a critical step, where EIA must be comprehensively integrated into decision-making processes, ensuring thorough consideration of environmental impacts and alternatives (Naser, 2015). Gulf countries should strengthen EIA legislation, including detailed procedural guidelines for assessments, public participation, and monitoring of ecological impacts. This can be achieved by adopting a strategic approach to environmental assessment, which accounts for cumulative effects of multiple projects and involves stakeholders in a more meaningful way, thereby improving water quality management and preserving key ecosystems. Furthermore, establishing a legal framework mandating spatial mapping of sensitive coastal and marine environments would aid in identifying and protecting vulnerable areas from the adverse effects of reclamation activities. By doing so, Gulf countries could ensure a more sustainable approach to development, where the preservation of biodiversity and ecological integrity is given equal importance as economic growth.*

Improving the design of artificial structures is another avenue to reduce their environmental impact. Artificial structures built on land reclamation should include open channels allowing ocean currents to partially flow through the structure. This permeability would facilitate the flushing of the area downstream and hence improve its water quality (Jiang et al., 2020). Very Large Floating Structures (VLFS) also present an interesting alternative to traditional land reclamation methods, especially in terms of reducing their impact on ocean currents dynamics and subsequently on water quality (Wang and Tay, 2011). They consist in expansive, man-made platforms that float on the water's surface and have been designed for various purposed such as airports, hotels, or agricultural space (Lamas-Pardo et al., 2015). While they are most cost-effective in areas where water depth is significant and might thus not be suitable everywhere in the Gulf, their environmental im-

pact is much lower than land reclamation. VLFS do not create physical barriers that could alter ocean currents, which is crucial for maintaining water quality. Furthermore, they do not require the installation of permanent structures over the sea bed, thus preserving benthic habitats. They can further be dismantled if the sea area is needed for other purposes in the future.

As with any modelling study, it is important to acknowledge the limitations and assumptions inherent to our approach. Firstly, our model focuses solely on ocean currents, excluding wind-generated waves. Consequently, we do not account for transport pattern alterations resulting from wave deflection by artificial structures. The impact of waves on transport processes, particularly through the Stokes drift, can be significant for sediment erosion and accretion near structures built perpendicular to the shoreline (Walker, 2012). The influence of Stokes drift on pollutant transport dynamics in Doha Bay is however expected to be limited. On the one hand, the narrow and curved configuration of the Strait of Hormuz prevents swell waves from penetrating into the Gulf, leading to a wave climate in both the Gulf and Doha Bay that is predominantly shaped by locally generated wind waves (Beyramzadeh et al., 2021). On the second hand, the bay is sheltered from the prevailing southeastward Shamal winds and wave growth is thus fetch-limited as the winds move off the land and back onto the water (Liao and Kaihatu, 2016). Another assumption is that our model employs a two-dimensional barotropic approach, meaning that the vertical ocean circulation dynamics are neglected. This assumption is supported by Doha Bay's shallow depth and significant tidal amplitude. Lastly, we use residence time as a surrogate for water quality. This is an approximation, as water quality is affected by various pollutants, each undergoing distinct chemical reactions like hydrolysis, oxidation-reduction, photolysis, biotic transformation, adsorption, among others (Lyman, 2020). In this study, we implicitly assume that these complex chemical processes occur more slowly than the bay's flushing by ocean currents. Thus, in the short term, flushing is presumed to be the primary determinant of water quality in the bay.

Reviewer #2

(1) *Abstract: the section presents a good synopsis of the work but could have also use extra sentences to summarize the main results found - although some of these were mentioned in the author's summary.*

Response: We followed the reviewer's suggestion and enhanced the abstract by incorporating an additional sentence. This sentence succinctly summarizes our findings on the alterations in ocean circulation patterns attributable to the land reclamation projects in the northern part of the Bay, which were not mentioned before (see line xx).

It reads: *Our findings reveal that the residual ocean circulation in Doha Bay was predominantly impacted by northern developments post-2000. Between 1980 and 2000, the bay's residence time saw a modest rise, of about one day on average.*

(2) *Introduction: this sets the scene smoothly well, but the justification of the research could have been more robust - perhaps adding a literature review section.*

Response: Thank you for your constructive feedback on the introduction of our paper. While the primary focus of our study is not to deliver an exhaustive review of the environmental impacts of land reclamation projects, we acknowledge the importance of a robust justification for our

research. This point was already partly addressed in the first paragraph of the introduction (see lines 2-15) that was exposing the broad environmental issues related to land reclamation. We have nonetheless taken the reviewer's comment into account by adding more specific examples of the detrimental environmental impact of land reclamation at the end of the third paragraph (see lines 40-43).

The additional text reads: *More broadly, several studies have underscored the detrimental impacts of land reclamation on water quality, particularly when carried out without comprehensive planning. Notable examples include reclamation projects in Qinzhou and Bohai Bays in China (Lyu et al., 2022; Zhang et al., 2023), and along the Shihwa coast in the Republic of Korea (Lee et al., 2014).*.

(3) *Methodology: A systematic research method has been used or described. What is not clear is why is this method the best to achieve the aim? Can these lead to generalisable conclusions?*

Response: Thank you for your inquiry regarding the methodology of our study. As detailed in the Methods section, we have computed the residence time as it is a simple and efficient metric to assess the flushing capacity of the bay. Flushing capacity is crucial for understanding the removal dynamics of waterborne pollutants by ocean currents, thereby providing insights into the duration these pollutants remain within the bay's ecosystem. The relevance of residence time as a metric becomes particularly pronounced in scenarios where the specific nature and sources of pollutants are not well-defined. It serves as an effective tool for evaluating environmental changes driven by external factors, such as alterations in coastline topography, which is the focus of our study. Regarding the sufficiency of our methodological justification, we believe that the explanations provided in both the Introduction (see lines 44-58) and the Methods (see lines 143-177) sections adequately support our chosen approach. As for the question of generalizability, our study's conclusions are indeed specific to Doha Bay and are not intended to be universally applicable to all land reclamation projects. However, it is worth noting that similar methodologies applied to other artificial structures, as shown in studies like Cavalcante et al. (2012) for The Palm in Dubai, have yielded findings that align with ours. This suggests that while our conclusions are specific, the underlying principles and outcomes observed in our study may have broader relevance in similar environmental contexts.

(4) *Results/Discussion/Conclusion: Good work but the result should be able to show clearly what is new? what is surprising? What was learn when compared to the existing body of knowledge? What are the limitations and novelty of this paper?*

Response: Thank you for your encouraging feedback! In response to your suggestions, we have taken steps to more explicitly articulate the main contributions and findings of our study in the Discussions and Conclusions section. In the opening paragraph of this section, we now provide a concise summary of our main findings, emphasizing the significant increase in water residence time along Doha's coastline over the past 40 years, primarily attributable to the land reclamation projects in the northern part of the Bay. We delve into how this increase is intricately linked to alterations in local ocean circulation patterns caused by the artificial structures, leading to a reduced flushing capacity of the bay. While we believe that our discussion effectively underscores

the novelty of our research, we acknowledge your point regarding the need to more thoroughly address the study's limitations. To this end, we have introduced an additional paragraph in the discussion section, where we discuss the limitations inherent to the models we employed and the constraints of using water residence time as a sole diagnostic for water quality. This new addition, found at lines 356-377, aims to provide a balanced view of our study, acknowledging its constraints while highlighting its innovative aspects. Through these enhancements, we hope to have clarified what sets our research apart, what new insights it brings to the field, and how it contributes to the existing body of knowledge, all while transparently acknowledging its limitations.

The new paragraph reads: *As with any modelling study, it is important to acknowledge the limitations and assumptions inherent to our approach. Firstly, our model focuses solely on ocean currents, excluding wind-generated waves. Consequently, we do not account for transport pattern alterations resulting from wave deflection by artificial structures. The impact of waves on transport processes, particularly through the Stokes drift, can be significant for sediment erosion and accretion near structures built perpendicular to the shoreline (Walker, 2012). The influence of Stokes drift on pollutant transport dynamics in Doha Bay is however expected to be limited. On the one hand, the narrow and curved configuration of the Strait of Hormuz prevents swell waves from penetrating into the Gulf, leading to a wave climate in both the Gulf and Doha Bay that is predominantly shaped by locally generated wind waves (Beyramzadeh et al., 2021). On the second hand, the bay is sheltered from the prevailing southeastward Shamal winds and wave growth is thus fetch-limited as the winds move off the land and back onto the water (Liao and Kaihatu, 2016). Another assumption is that our model employs a two-dimensional barotropic approach, meaning that the vertical ocean circulation dynamics are neglected. This assumption is supported by Doha Bay's shallow depth and significant tidal amplitude. Lastly, we use residence time as a surrogate for water quality. This is an approximation, as water quality is affected by various pollutants, each undergoing distinct chemical reactions like hydrolysis, oxidation-reduction, photolysis, biotic transformation, adsorption, among others (Lyman, 2020). In this study, we implicitly assume that these complex chemical processes occur more slowly than the bay's flushing by ocean currents. Thus, in the short term, flushing is presumed to be the primary determinant of water quality in the bay.*.

(5) *Discussion and conclusion - perhaps should be separated and conclusion should be rewritten to show whether the aim was achieved and identify the contribution to knowledge.*

Response: Thank you for your suggestion regarding the structure of the Discussion and Conclusions section. We have considered your advice carefully and, while we appreciate the rationale behind separating these sections, we have decided to maintain a combined Discussion and Conclusions section. This decision is made to ensure a cohesive and integrated presentation of our findings, thereby avoiding potential redundancies. In our revised section, we have structured the content to ensure clarity and coherence. The section begins with a concise summary of the main findings, directly addressing whether the aims of the study were achieved and highlighting our contributions to the existing body of knowledge. This is followed by an expanded discussion that contextualizes our findings within the broader scope of similar land reclamation projects in the Gulf region. Furthermore, we have included recommendations for mitigating the environmental impacts of artificial structures, which not only adds practical value to our study but also aligns with the broader goals of sustainable development. We then move on to discuss the limitations of

our current modeling approach, particularly in using residence time as a proxy for water quality, providing a balanced and transparent evaluation of our research. Finally, the section concludes with forward-looking perspectives, contemplating the potential implications of future coastline development in Doha and the influx of visitors for major international events on the bay's water quality. This holistic approach ensures that our Discussion and Conclusions section effectively encapsulates the essence of our research, its significance, and its potential future implications.

Journal requirements

(1) *Please ensure that your manuscript meets PLOS ONE's style requirements, including those for file naming.*

Response: We have tried, throughout the paper, to take all PLOS ONE's style requirements into account.

(2) *Acknowledgments and funding statement*

Response: As requested, we removed the funding statement from the acknowledgments. The current Funding Statement is fine and should not be updated.

(3) *Map/satellite images in Fig 1, 2, 3 and 4.*

Response: All the maps shown in Fig. 1, 2, 3 and 4 were produced with data from OpenStreepMap. OpenStreetMap is open data, licensed under the Open Data Commons Open Database License (ODbL) by the OpenStreetMap Foundation (OSMF). Data users are free to copy, distribute, transmit and adapt our data, as long as OpenStreetMap and its contributors are credited (see `https://www.openstreetmap.org/copyright/en`). We have therefore added the following sentence to the captions of all 4 figures: *The base map was sourced from OpenStreetMap and OpenStreetMap Foundation. This figure contains information from OpenStreetMap and OpenStreetMap Foundation, which is made available under the Open Database License (ODbL).*

(4) *Supplementary figure*

Response: We have amended the supplementary figure file type to "Supporting Information".

(5) *Captions for Supporting Information files*

Response: The caption of the Supporting Information file has been included as requested.

(6) *Reference list*

Response: We have checked the reference list to ensure that it is correct and up-to-date.

References

Beyramzadeh, M., Siadatmousavi, S.M., Derkani, M.H., 2021. Calibration and skill assessment of two input and dissipation parameterizations in WAVEWATCH-III model forced with ERA5 winds with application to Persian Gulf and Gulf of Oman. Ocean Engineering 219, 108445.

Cavalcante, G.H., Kjerfve, B., Feary, D.A., 2012. Examination of residence time and its relevance to water quality within a coastal mega-structure: The Palm Jumeirah Lagoon. Journal of Hydrology 468, 111–119.

Fischer, H.B., 1979. Mixing in inland and coastal waters. Academic press.

Hanert, E., Mohammed, A.V., Veerasingam, S., Dobbelaere, T., Vallaeys, V., Vethamony, P., 2023. A multiscale ocean modelling system for the central Arabian/Persian Gulf: From regional to structure scale circulation patterns. Estuarine, Coastal and Shelf Science 282, 108230.

Jiang, W., Zhang, H., Peng, J., Song, C., Li, X., 2020. Waterway design criteria of land reclamation based on an environmental fluid dynamics computer model. Journal of Coastal Research 108, 89–94.

Lamas-Pardo, M., Iglesias, G., Carral, L., 2015. A review of very large floating structures (VLFS) for coastal and offshore uses. Ocean Engineering 109, 677–690.

Lee, C.H., Lee, B.Y., Chang, W.K., Hong, S., Song, S.J., Park, J., Kwon, B.O., Khim, J.S., 2014. Environmental and ecological effects of Lake Shihwa reclamation project in South Korea: A review. Ocean & coastal management 102, 545–558.

Liao, Y.P., Kaihatu, J.M., 2016. Numerical investigation of wind waves in the Persian Gulf: Bathymetry effects. Journal of Atmospheric and Oceanic Technology 33, 17–31.

Lyman, W., 2020. Transport and transformation processes, in: Fundamentals of Aquatic Toxicology. CRC Press, pp. 449–492.

Lyu, H., Song, D., Zhang, S., Wu, W., Bao, X., 2022. Compound effect of land reclamation and land-based pollutant input on water quality in Qinzhou Bay, China. Science of The Total Environment 826, 154183.

Monsen, N.E., Cloern, J.E., Lucas, L.V., Monismith, S.G., 2002. A comment on the use of flushing time, residence time, and age as transport time scales. Limnology and oceanography 47, 1545–1553.

Naser, H.A., 2015. The role of environmental impact assessment in protecting coastal and marine environments in rapidly developing islands: The case of Bahrain, Arabian Gulf. Ocean & Coastal Management 104, 159–169.

Rao, P.G., Hatwar, H., Al-Sulaiti, M.H., Al-Mulla, A.H., 2003. Summer Shamals over the Arabian Gulf. Weather 58, 471–478.

Takeoka, H., 1984. Fundamental concepts of exchange and transport time scales in a coastal sea. Continental Shelf Research 3, 311–326.

Walker, H.J., 2012. Artificial structures and shorelines. volume 10. Springer Science & Business Media.

Wang, C., Tay, Z., 2011. Very large floating structures: Applications, research and development. Procedia Engineering 14, 62–72.

Zhang, H.X., Shen, Y.M., Tang, J., 2023. Numerical investigation of successive land reclamation effects on hydrodynamics and water quality in Bohai Bay. Ocean Engineering 268, 113483.
